# Endothelial Protein C Receptor: A Multifunctional Mediator in the Pathophysiology of Rheumatoid Arthritis

**DOI:** 10.3390/cells14070485

**Published:** 2025-03-24

**Authors:** Meilang Xue, Lyn March

**Affiliations:** 1 Sutton Arthritis Research Laboratory, Sydney Musculoskeletal Health, Kolling Institute, Faculty of Medicine and Health, The University of Sydney, Sydney, NSW 2065, Australia; 2 The Australian Arthritis and Autoimmune Biobank Collaborative (A3BC), Sydney Musculoskeletal Health, Kolling Institute, Faculty of Medicine and Health, The University of Sydney, Sydney, NSW 2065, Australia; lyn.march@sydney.edu.au; 3 Department of Rheumatology, Royal North Shore Hospital, Syndey, NSW 2065, Australia

**Keywords:** endothelial protein C receptor, rheumatoid arthritis, inflammation, coagulation, endothelial barrier, synovial fibroblasts

## Abstract

The endothelial protein C receptor (EPCR) is gaining recognition for its diverse functions that extend beyond its traditional role in the protein C anticoagulant pathway. This comprehensive review examines how EPCR contributes to the pathophysiology of rheumatoid arthritis (RA), an autoimmune disorder characterized by persistent inflammation and joint destruction. We explore how EPCR influences inflammatory responses and the coagulation cascade, affects endothelial function and vascular integrity, and regulates the characteristics of synovial fibroblasts in the context of RA. Furthermore, the review highlights the mechanisms by which EPCR affects disease progression, its potential use as a biomarker for disease activity, and the therapeutic implications of targeting EPCR in the treatment of RA. By synthesizing current research findings, this review aims to provide a detailed understanding of EPCR’s role in RA, offering insights into innovative diagnostic and therapeutic strategies that could improve patient outcomes.

## 1. Introduction

Rheumatoid arthritis (RA) is a chronic inflammatory disorder that primarily affects the joints, resulting in progressive inflammation of the synovial membrane, destruction of cartilage, erosion of bone, and, ultimately, the loss of joint functionality [1,2]. Additionally, RA may manifest various systemic effects that can impact the overall well-being of individuals [3]. If left untreated, RA can cause severe disability and decreased quality of life. The endothelial protein C receptor (EPCR, CD201) is a transmembrane glycoprotein encoded by the *PROCR* gene. Initially discovered on endothelial cells, EPCR binds to activated protein C (APC) and its precursor protein C (PC), regulating APC’s anticoagulant, anti-inflammatory, anti-apoptotic, and barrier-protective functions [4]. EPCR is now known to be a versatile receptor expressed by many cell types, interacting with multiple ligands and performing diverse functions [5]. This review explores EPCR’s role in RA, focusing on its involvement in immune response, coagulation, synovial fibroblast function, and potential therapeutic applications.

## 2. EPCR and Its Structure

The *PROCR* gene is located on chromosome 20q11.2, spanning 6kD with four exons [6,7]. The mature protein produced by this gene is a 46-kD type I transmembrane protein with 221 amino acids, including an extracellular domain, a transmembrane domain, and a short cytoplasmic tail [8]. The extracellular domain, featuring two antiparallel alpha-helices above an eight-stranded beta-sheet, is crucial for binding phospholipids, maintaining receptor integrity, and interacting with ligands [9].

EPCR exhibits structural similarities with the major histocompatibility complex (MHC) class I and the CD1 family of receptors, enabling it to bind and present ligands similarly to how MHC molecules present antigens [5,9]. The membrane-anchored form of EPCR (mEPCR) has the ability to internalize, recycle, and return to the cell surface, renewing its ligand load and influencing immune responses [10,11]. Additionally, EPCR may act as an autoantigen, attracting autoantibodies and affecting inflammatory response in autoimmune diseases. Moreover, studies have demonstrated that EPCR can bind to various lipids [12,13,14], which impacts its anticoagulant properties and the development of autoimmune disorders.

The membrane-anchored EPCR can be cleaved to produce soluble (s)EPCR through proteolytic ectodomain shedding and EPCR mRNA alternative splicing. The main protease responsible for ectodomain shedding is the metalloprotease tumor necrosis factor-α-converting enzyme (TACE/ADAM17), which cleaves EPCR near the cell membrane [15,16]. This shedding process occurs rapidly and is regulated by various signaling pathways and inflammatory stimuli, including TNF-α and lipopolysaccharide [15,16]. Alternatively, sEPCR can be generated more slowly through mRNA splicing, leading to a truncated version secreted into the plasma. This slower process is particularly more common in individuals with the haplotype 3 (H3) of the *PROCR* gene [15,16]. While sEPCR competes with mEPCR for binding sites, it does not enhance the generation of APC [17] nor initiate downstream signaling as mEPCR does [5]. Increased levels of sEPCR may contribute to diseases characterized by excessive inflammation and coagulation abnormalities, such as RA [18,19].

Intracellular EPCR has been reported in endothelial [10] and epithelial [20] cells, mainly around the perinuclear region [10]. This protein can translocate into the nucleus, where it may impact gene expression by internalizing and transporting its ligands [10].

## 3. *PROCR* Gene Mutations

Mutations in the *PROCR* gene can affect the expression, function, and concentrations of sEPCR and mEPCR. Notable mutations include a 23 bp insertion in exon 3 (position 6367) [21] and various single-nucleotide polymorphisms (SNPs) in both coding and non-coding regions. The 23 bp insertion leads to a truncated protein lacking parts of the extracellular domain, transmembrane domain, and cytoplasmic tail. As a result, the protein can not localize to the cell surface, be secreted, or bind to APC [17]. This particular mutation occurs in 0.48% of patients with venous thromboembolism (VTE) and 0.38% of healthy controls [17], suggesting that it is likely a risk factor for VTE.

More than sixteen SNPs have been identified in the *PROCR* gene, which can be categorized into four main haplotypes (H1–H4) [7]. A haplotype is a set of linked SNP alleles that typically occur together. Identifying specific alleles of a haplotype can aid in locating other nearby polymorphic sites. H1, H3, and H4 contain haplotype-specific SNPs, while H2 contains the common allele of each SNP [17]. The frequencies of the haplotype-tagged rare alleles and the effects of these four haplotypes are detailed in Table 1.

Rare point mutations in the *PROCR* gene [32] and its promoter region [33] have also been reported. For example, the C2769T mutation causes an arginine-to-cysteine substitution at position 96 in the mature protein [32], though the effects of these mutations are not well understood.

EPCR’s complex architecture, including specific binding sites, MHC/CD1 homology, soluble and intracellular forms, and functional mutations, underscores its diverse roles in coagulation, inflammation, cellular protection, and homeostasis. A deeper understanding of these EPCR features is essential to unlocking its therapeutic potential in conditions marked by increased inflammation and coagulation, such as RA.

## 4. The General Functions of EPCR

Originally identified in endothelial cells, EPCR is now found to be expressed in a wide variety of cell types, including hematopoietic stem cells (HSCs) [34,35], monocytes [17], neutrophils [18], smooth muscle cells [19], keratinocytes [20], T cells [21], fibroblasts, cardiomyocytes [12], and even platelets. It plays a significant role in regulating anticoagulation, inflammation, and cell stemness.

Beyond its crucial interaction with PC and APC, EPCR can also bind to other ligands such as factor (F)VII, FX, the T cell receptor (TCR) [36], antiphospholipid antibodies (aPLs) [12], and secretory phospholipase A2 group V (sPLA2V) [37]. Table 2 lists these ligands and associated functions when bound to EPCR. This versatility positions EPCR as an important factor in shaping innate and adaptive immune responses, depending on the presence of different ligands. Furthermore, like the MHC/CD1 protein family [21], EPCR can independently influence innate and adaptive immune responses. Complete deficiency of EPCR in mice is lethal during embryonic development [38], emphasizing its essential functions in normal homeostasis.

### 4.1. Regulation of Blood Coagulation

EPCR can promote the generation of APC, which inactivates FVa and FVIIIa. By inactivating these factors, APC helps to reduce thrombin generation, thereby maintaining balance within the coagulation system [52]. Disruption of the *PROCR* gene can lead to fibrin accumulation and thrombosis in the placenta of mice [38], highlighting its importance in preventing excessive clot formation. Mice with an EPCR variant that cannot bind to PC demonstrate reduced PC activation and increased thrombin generation when exposed to thrombotic and inflammatory stimuli [53]. These findings indicate that EPCR is essential for maintaining equilibrium in the coagulation system and preventing abnormal clotting.

### 4.2. Regulation of Inflammation

EPCR’s effects on inflammation are significantly influenced by the specific ligands that interact with it. When EPCR binds to PC/APC, it activates cytoprotective signaling pathways that promote anti-inflammatory, anti-apoptotic, and barrier-stabilizing effects [40]. The interaction of EPCR with FVIIa also initiates anti-inflammatory and protective responses. This process relies on the engagement of protease-activated receptor-1 (PAR1) and β-arrestin-1 [42]. On the other hand, when plasmodium falciparum erythrocyte membrane protein 1 (PfEMP1) binds to EPCR, it enables parasite-infected red blood cells to adhere to blood vessel walls. This adherence leads to the disruption of the blood–brain barrier, increased vascular leakage, localized inflammation, and, ultimately, severe malaria [45]. Additionally, the binding of EPCR to aPLs has been associated with increased fetal loss and thrombosis in murine models [12]. Moreover, the interaction of EPCR with sPLA2V impairs the endothelial barrier [51] and enhances the invasiveness of RA synovial fibroblasts (RASF) [50].

EPCR also regulates inflammation independently of its interaction with ligands. For instance, it inhibits the generation of Th17 cells [54], and mice with a severe deficiency of EPCR are more susceptible to dextran sulfate sodium (DSS)-induced colitis, showing increased inflammation and compromised mucosal barrier function [55]. On the other hand, elevated levels of EPCR are associated with poor outcomes in colorectal and lung cancers [56,57], severe lung infection and inflammation [58], and a poor treatment response in patients with lupus nephritis [59]. In psoriasis patients, EPCR levels on circulating T cells positively correlate with disease severity, and anti-TNF treatment reduces both EPCR expression and disease severity [60]. Interestingly, EPCR deficiency offers protection against bacterial-induced lung injury [61], joint bleeding-induced inflammation [62], and collagen-induced arthritis (CIA) [63] in mice. This deficiency also limits the development of lupus and antiphospholipid syndrome (APS) [12], an autoimmune condition characterized by recurring thrombotic events in mice. APS is commonly observed in patients with lupus, RA, cancers, and many other autoimmune/inflammatory diseases.

The findings indicate that the role of EPCR in inflammation is both complex and dependent on the context. Depending on the specific ligand interactions and the cellular environment, EPCR can either inhibit or promote inflammation. This dual capacity enables EPCR to serve a versatile function in the body’s inflammatory responses, highlighting its potential as a therapeutic target for inflammatory and autoimmune disorders.

### 4.3. Regulation of Cell Stemness

EPCR is an important indicator in stem cell research, particularly for HSCs. It facilitates their isolation and characterization [64,65], enhances their self-renewal and quiescence, and ensures their long-term maintenance [65]. EPCR is likely identical to the intracellular murine protein CCD41 [66], which is essential for the survival and maintenance of HSCs. The lack of CCD41 leads to defects in HSC function, diminishing their capability to repopulate and maintain the hematopoietic system [67]. EPCR also serves as a stem cell marker for human epidermal keratinocytes [20], human cord blood HSCs [64], and progenitor cells of endothelium [68] and neurons [69]. In aggressive triple-negative breast cancer cells, EPCR expression is a characteristic of cancer stem cell-like populations with tumor-initiating properties in vivo [70]. Interestingly, EPCR’s functions in coagulation and inflammation are mainly controlled by EPCR on non-hematopoietic cells [71,72].

Overall, EPCR plays a crucial role in coagulation, inflammation, and stemness, highlighting its importance in maintaining health and preventing various pathophysiological conditions.

## 5. EPCR and Rheumatoid Arthritis

RA is a prevalent chronic inflammatory disease affecting 0.5–1% of the worldwide population, predominantly women. It can damage both joints and extra-articular organs [3]. Consequently, RA patients are more likely to develop comorbidities such as cardiovascular diseases (CVD) and VTE [73]. Without appropriate intervention, this condition can result in significant joint damage and increased risk for CVD and VTE, ultimately leading to disability and premature death.

The hallmark of RA is inflammation of the synovial membrane surrounding the joints [2]. This inflammation causes synovial hyperplasia and the formation of pannus [74], an abnormal layer of fibrovascular tissue that invades and damages cartilage and bone [1,2]. Immune cells such as T cells, B cells, macrophages, and neutrophils release pro-inflammatory cytokines like TNF-α, IL-1, and IL-6 and contribute to this process [1,2]. RA is also linked to specific autoantibodies, including rheumatoid factor (RF), anti-citrullinated protein antibodies (ACPA), and aPLs. These autoantibodies form immune complexes that further drive inflammation in RA [2].

### 5.1. Role of EPCR in RA

Recent studies reveal the significant role of EPCR in RA and suggest its potential as a biomarker for assessing disease activity and severity. Importantly, the effects of mEPCR and sEPCR appear to differ, indicating the necessity for additional investigation into their impacts on the disease’s pathophysiology.

#### 5.1.1. Cell Membrane-Anchored EPCR

**Figure 1:**
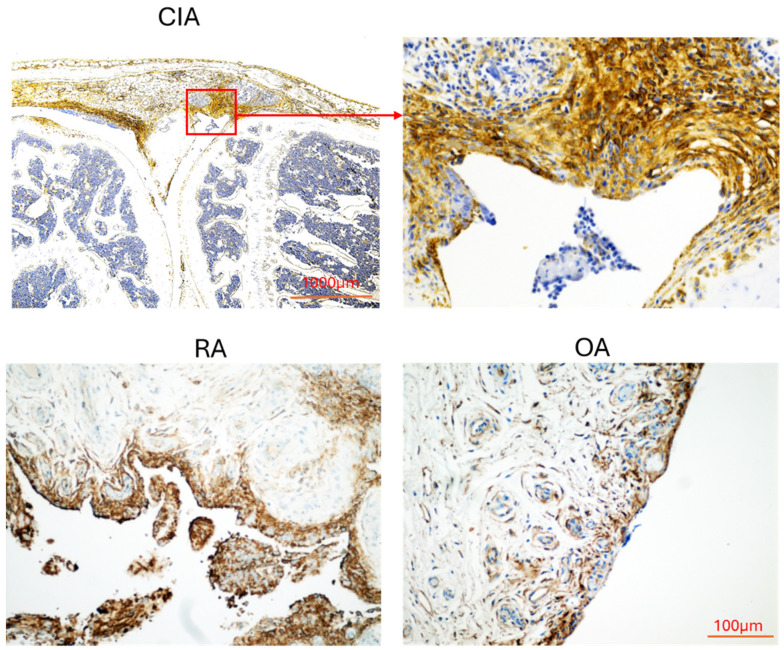
The expression of EPCR in synovial tissues. The expression of EPCR in synovial tissues from mice with collagen-induced arthritis (CIA) and patients with rheumatoid arthritis (RA) and osteoarthritis (OA). The brown color indicates EPCR staining.

This EPCR is present in the synovial tissue of the joint but is notably overexpressed in RA, especially in the synovial lining layer (Figure 1) [37,50,75]. Various immune cell types, including dendritic cells (DC), monocytes, natural killer (NK) cells, B cells, T cells, and neutrophils in RA, also express EPCR [19,76]. Elevated levels of EPCR on circulating T cells and NK cells demonstrated a negative relationship with RA disease activity measures [19,50]. In contrast, one study found that the percentage of circulating CD4+EPCR+ T cells in RA patients was lower than that in individuals with osteoarthritis (OA) [18]. Decreasing EPCR expression in RASF resulted in lower viability, invasiveness, and the production of inflammatory mediators by these cells [37]. In CIA, mice lacking EPCR severely exhibited milder arthritis and lower frequencies of Th1 and Th17 cells in synovial tissues [63]. Conversely, EPCR and PC/APC were observed to co-localize in RA monocytes /macrophages, and APC enhances the expression of EPCR in these cells, which subsequently increases its ability to inhibit monocyte activation and migration [75]. In the CIA, APC alleviates the severity of arthritis through its interaction with EPCR [50]. Furthermore, APC can inhibit NETosis, a process that contributes to synovial inflammation and joint damage in RA [77], partially via signaling pathways associated with EPCR [78].

#### 5.1.2. Soluble EPCR

RA patients have higher levels of plasma sEPCR compared to healthy controls. These elevated sEPCR levels are positively associated with inflammatory markers such as IL-6, IL-17, ACPA, and RF [17,18]. However, they do not correlate with RA disease activity measures like DAS28 [19]. Furthermore, the administration of sEPCR has been shown to induce disease remission in CIA mice by inhibiting Th17 differentiation [17]. sEPCR is also present in synovial fluid, but no significant differences are observed between OA and RA [20].

#### 5.1.3. *PROCR* Gene Variants

Variants of the *PROCR* gene significantly influence the expression and function of EPCR, potentially playing a critical role in the pathogenesis of RA. Specifically, the SNP H3 G phenotype affects both sEPCR and mEPCR levels [26], which in turn modulate APC and FVII generation [26], impacting the inflammatory response and the disease progression [17,18]. Research indicates that this SNP is negatively correlated with measures of disease activity in RA [19]. Patients with RA have an elevated risk of developing VTE and CVD [73]. The SNP H3 G phenotype is associated with a higher risk of VTE [27,79] while being linked to a lower risk of coronary artery disease and myocardial infarction [27,79]. In contrast, the SNP H1 G/C genotype is related to increased plasma APC levels and reduced plasma sEPCR levels [79,80], which appear to protect against VTE in patients with APS [26].

### 5.2. Potential Action Mechanisms of EPCR in RA

The role of EPCR in RA is likely shaped by the specific ligands it binds to and the characteristics of the receptor itself. Various ligands, such as PC/APC, FVII, aPLs, sPLA2V, and TCR, are commonly found in RA and can impact EPCR’s role in inflammation and coagulation. These ligand-specific interactions allow EPCR to have a range of effects on immune responses and disease progression in RA. Additionally, the presence of sEPCR further influences its function in this condition.

#### 5.2.1. Ligand-Specific Functions of EPCR in RA

Activated protein C

The interaction between EPCR and APC leads to the activation of PAR1. This activation inhibits pro-inflammatory cytokines such as TNF-α, IL-6, and IL-1β, reduces the adhesion of leukocytes to the endothelium, and decreases the infiltration of inflammatory cells into tissues and increases anti-inflammatory cytokines, therefore mitigating the inflammatory response [81]. These anti-inflammatory effects are particularly important in RA.

In RA, synovitis occurs due to leukocytes infiltrating the synovium. This infiltration results from leukocytes migrating from distant sites in response to adhesion molecules and chemokines expressed by activated endothelial cells in synovial microvessels [2]. EPCR-APC signaling enhances the endothelial barrier function, reducing vascular permeability and preventing inflammatory cells from leaking into the joint, which reduces synovial inflammation and tissue damage [37]. The data show that APC inhibits the migration and activation of RA monocytes and prevents inflammatory arthritis in mice via binding to EPCR [75].

Additionally, endothelial dysfunction in RA affects both large and small blood vessels, contributing to the increased cardiovascular risk in these patients [50]. Improving endothelial function through EPCR-APC signaling could help mitigate this increased cardiovascular risk.

Factor VII

FVII initiates the coagulation cascade by forming a complex with tissue factor, which activates FIX and FX, leading to blood clot formation [82]. In RA, increased vascular permeability and endothelial dysfunction can raise tissue factor levels, enhancing its interaction with FVII and promoting coagulation [83]. These tissue factor/FVII complexes, often found in RA joints, may significantly contribute to chronic destructive arthritis [84].

EPCR may help balance coagulation and inflammation in RA through its interaction with FVII. When FVIIa binds to EPCR, it activates protective signaling pathways in endothelial cells, reducing vascular permeability and inflammation [42]. Studies suggest that FVIIa binding to EPCR aids its transport from blood to extravascular tissues, promoting EPCR endocytosis and helping clear FVIIa from circulation [10]. However, this interaction prolongs the presence of FVIIa in extravascular tissues and downregulates the PC anticoagulation pathway, promoting clot formation [41]. In a mouse hemophilia model, the hemostatic effect of recombinant FVIIa is achieved by downregulating the EPCR-mediated anticoagulant pathway. Blocking EPCR alone does not reduce bleeding severity in hemophilic mice. This indicates that the primary mechanism through which FVIIa interacts with EPCR involves downregulating APC generation [62]. Therefore, the interaction between FVII and EPCR has dual effects: its anti-inflammatory properties are beneficial, but its pro-coagulation effects can be detrimental in RA.

Secretory phospholipase A2 group V

sPLA2V plays a crucial role in lipid metabolism and inflammatory processes [85]. Its levels are significantly elevated in RA joints [86,87], where it promotes synovial cell growth and joint damage [87,88]. Higher levels of sPLA2V are also linked to faster arterial thrombosis [89].

Identifying sPLA2V as a ligand for EPCR adds complexity to our understanding of EPCR’s role in RA. This ligand competes with PC and APC for the same binding site on EPCR [37], disrupting normal APC-EPCR interactions. In mouse models, overproduction of sPLA2V significantly reduces APC levels [89]. sPLA2V-EPCR complex promotes the growth and cartilage degrading potential of RASF [50] and compromises the integrity of the endothelial barrier [51].

Antiphospholipid antibodies

aPLs are found in 5–75% of RA patients and are linked to an increased risk of arterial thrombosis [90]. When aPLs bind to cell surface receptors, such as β2-glycoprotein I, they induce the internalization of the antibody–receptor complex through endocytosis [91]. Inside the cell, aPLs activate various intracellular signaling pathways that promote pro-thrombotic and inflammatory responses, leading to tissue damage [91].

The binding of aPLs to EPCR inhibits its interaction with APC, reducing the anti-inflammatory and anticoagulant effects of the PC/APC pathway. This binding also facilitates the internalization of aPLs by immune cells, resulting in the activation of monocytes, DC, and trophoblast cells. Consequently, this interaction intensifies the pro-inflammatory and pro-thrombotic environment in RA patients with aPLs, exacerbating vascular complications and worsening joint inflammation. Research suggests that specific targeting EPCR signaling can mitigate the effects of aPLs and reduce autoimmunity in mouse models of systemic lupus erythematosus [12]. Given these insights, monitoring the levels of EPCR and aPLs in RA patients can be vital for developing effective treatment strategies to manage inflammation and prevent complications. It is important to emphasize that while aPLs are clinically relevant, they are not specific to RA and are not incorporated into its diagnostic criteria.

T cell receptor

Willcox and his team found that EPCR functions as a ligand for the TCR in a specific subset of Vδ2− γδ T cells [36]. These γδ T cells, abundant in intestinal tissue [92], play a pivotal role in maintaining the balance between host and microbiota at the intestinal mucosal surface [93,94]. They detect invading bacteria through interactions with nearby epithelial cells, protecting against bacterial infiltration, especially during the critical initial hours following exposure [93,94]. The absence of these γδ T cells correlates with increased levels of resident microbiota in the small intestine [95], potentially leading to systemic inflammation.

The idea that RA originates from mucosal sites is supported by current findings: the presence of ACPA in the lungs, interstitial inflammatory changes in individuals at risk for RA, the association of periodontitis with RA, and the dysbiosis of gut microbiomes in patients who have recently developed RA [96]. Previous studies indicate that the progressive loss of the PC-EPCR pathway in gastrointestinal disorders, such as Crohn’s disease and ulcerative colitis, is linked to inflammation, compromised mucosal barrier function, and disease severity [97,98]. However, the mechanisms by which EPCR interacts with these cells in the mucosal sites are yet to be fully understood.

Moreover, growing evidence suggests that γδ T cells are directly involved in RA [99]. In RA, synovial effusions and membranes show a high concentration of γδ TCR-expressing T cells [100]. Their presence is associated with increased tissue inflammation and production of pro-inflammatory cytokines [101]. Furthermore, γδ T cells also interact with other immune cells, amplifying the overall inflammatory response and potentially leading to more severe joint damage [99]. Preventive depletion of γδ T cells in CIA has demonstrated a reduction in disease severity in DBA1/J mice [102]. Nonetheless, the relationship between EPCR and γδ T cell TCRs, along with the mechanisms by which this interaction regulates inflammation in RA, remains unclear.

#### 5.2.2. Coagulation Cascade and EPCR in RA

The coagulation cascade involves a series of enzymatic reactions that lead to blood clot formation, which is essential for stopping bleeding and initiating wound healing. However, it is crucial to balance clot formation and dissolution during inflammation. EPCR helps maintain this balance by promoting the anticoagulant pathway, particularly during inflammatory episodes [38,53]. This function prevents excessive clotting and ensures proper blood flow.

In RA, chronic inflammation can trigger the coagulation cascade, leading to a hypercoagulable state where blood is more likely to clot [103]. This imbalance between coagulation and anticoagulation is probably due to decreased levels of APC and increased levels of inflammatory cytokines and sEPCR, which reduce APC availability. As a result, patients with RA face a higher risk of developing VTE. Strong anti-inflammatory treatments such as Disease-Modifying Anti-Rheumatic Drugs (DMARDs) or APC can help alleviate this pro-thrombotic state, reducing the risk of blood clots and improving overall health outcomes [104].

#### 5.2.3. Modulation of T Cell Functions

RA is an autoimmune disease driven by T cells, where imbalances in both the proportions and functions of T cell subsets significantly contribute to its development [105]. EPCR is notably present in T cells [18,19] and regulates their activation and differentiation [54,106]. Th1 and Th17 cells, along with their associated effector cytokines, can trigger the onset of RA [105]. Studies have demonstrated that a lack of EPCR decreases synovial Th1 and Th17 cells and inhibits inflammatory arthritis in mice by suppressing T cell and DC activation and migration [63]. Conversely, in a mouse model of experimental autoimmune encephalomyelitis, a specific deficiency of EPCR in T cells has been linked to more Th17 cells and severe disease outcomes [54]. This suggests that EPCR’s role in T cells varies depending on the tissue or disease context.

#### 5.2.4. Regulation of RA Synovial Fibroblast Activity

RA is characterized by synovial inflammation and hyperplasia. In a healthy joint, the synovium provides lubrication and nutrients. However, in RA, the synovium transforms into an inflammatory mass known as pannus, which invades and erodes the surrounding cartilage and bone [107,108]. The primary culprits within the pannus are the activated RASF, also known as RA synoviocytes [109]. These cells are hyperproliferative, hyperinvasive, and resistant to apoptosis. They can produce inflammatory cytokines such as IL-6 and TNF-α and interact with immune cells, contributing to the maintenance and progression of RA [107,108]. Additionally, RASF can convert T regulatory cells into Th17 cells [110] and exhibit aggressive behaviors even without inflammation [111]. The in vitro invasiveness of RASF is closely linked to clinical bone erosion in RA patients [112]. Mice lacking synoviocytes are protected from cartilage damage induced by RA [113].

EPCR is highly expressed in the synovial lining cells of both human and animal models of RA (Figure 1). Inhibition of EPCR expression has been shown to reduce the proliferation, migration/invasion, and cartilage degradation ability of RASF in vitro [37]. Mice with a severe deficiency in EPCR exhibit reduced synovial hyperplasia in CIA [63] and in a mouse model of hemophilic arthropathy [62]. Therefore, EPCR likely plays a significant role in mediating the aggressive behaviors of RASF, similar to those observed in aggressive breast cancer [70] and lung cancers [57].

#### 5.2.5. sEPCR

The binding of sEPCR to PC/APC restricts the generation and functionality of APC, potentially increasing the risk of thrombosis [75] and inflammation. Elevated levels of sEPCR may also have procoagulant and pro-inflammatory effects, contributing to the inflammatory process in RA [18,19].

Conversely, one study showed that higher levels of sEPCR can stabilize and prolong the presence of PC and APC in the circulation by binding to them, preventing their rapid clearance from the bloodstream [15]. This helps maintain the anticoagulant and cytoprotective functions of APC for a longer duration, which is particularly important given APC’s short half-life of approximately 15 min [114].

**Figure 2:**
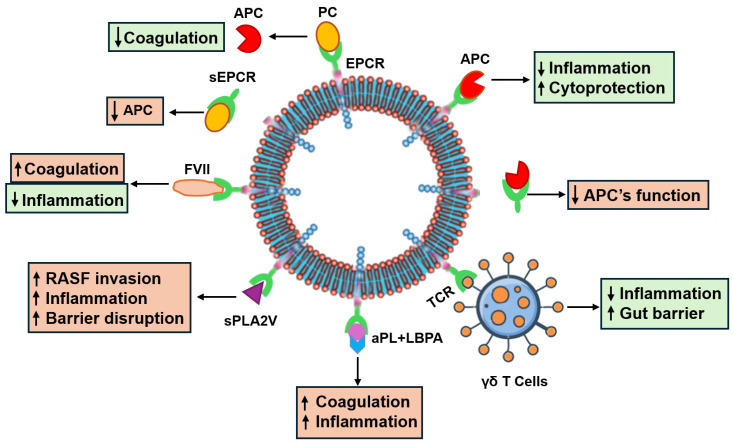
The complex functions of EPCR in rheumatoid arthritis. APC: activated protein C; aPL: antiphospholipid antibodies; FVII: factor VII; LBPA: lysophosphatidic acid; mEPCR: membrane-anchored endothelial protein receptor; PC: protein C; sEPCR: soluble EPCR; sPLA2: secretory phospholipase A2 group V; TCR: T cell receptor.

In summary, current research reveals a paradoxical role for EPCR in RA. While it exhibits anti-inflammatory and immunomodulatory properties, potentially offering protective effects, it can also contribute to tissue damage and exacerbation of joint inflammation. This dual functionality necessitates further investigation to elucidate the precise mechanisms by which EPCR influences RA pathogenesis (Figure 2).

## 6. Targeting EPCR Pathways for RA Treatment

### 6.1. Potential Therapeutic Strategies

Targeting the EPCR pathways offers a promising approach for treating RA due to EPCR’s role in modulating inflammation, barrier function, coagulation, and RASF. Several potential therapeutic strategies can be explored.

APC Therapy: Recombinant APC and its analogs have been developed to enhance the anti-inflammatory and cytoprotective effects mediated by EPCR. Human recombinant APC has been shown to reduce inflammation and protect joint tissues from damage in a mouse model of RA [50]. To avoid the potential bleeding risk of APC, mutant forms of APC, such as 3K3A-APC, which retains less than 10% of APC’s anticoagulant activity but preserves its anti-inflammatory and cytoprotective function, along with small peptides that mimic APC’s functions, have been created. These APC analogs have been used for preclinical studies and clinical trials in the treatment of wound healing [115], stroke [116], and cancer [117]. They also hold potential for use in RA treatment.

EPCR blocking antibody: EPCR blocking antibodies have shown promise in the treatment of conditions such as hemophilic arthropathy [62] and aPL-induced pathologies [12]. Although the extraembryonic expression of EPCR may be critical for embryo viability [118], mice with a significant shortage of EPCR remain healthy, exhibiting no signs of bleeding, spontaneous thrombosis, or inflammation [62,118]. Additionally, these mice are protected from inflammatory arthritis [63]. Therefore, inhibiting EPCR with blocking antibodies could be a viable therapeutic approach for RA.

Combination Therapies: Investigating the potential of combining EPCR-targeted therapies with existing RA treatments to enhance their efficacy and reduce side effects. This approach could provide a more comprehensive treatment strategy for RA.

Personalized Medicine: By incorporating EPCR-related biomarkers and genetic information, such as SNP profiles, into customized diagnostic and treatment plans for RA patients [19], therapies can be tailored to each individual’s EPCR profile. This personalized approach can enhance treatment outcomes and minimize adverse effects.

These strategies highlight the potential of targeting EPCR pathways to develop innovative and effective treatments for RA. Continued research and clinical trials will be crucial in identifying the most effective approaches and advancing these therapies toward clinical application.

### 6.2. Potential Risks and Mitigation Strategies

Targeting EPCR in RA carries potential risks due to its various functions, which include the following: (1) disruption of coagulation (leading to thrombosis/bleeding); (2) impaired vascular integrity (resulting in edema, inflammation, or cardiovascular comorbidities); (3) unintended modulation of inflammatory pathways; (4) disrupted stem cell regulation (affecting hematopoiesis, tissue repair); and (5) unexpected interactions with ligands.

To mitigate these risks, the following strategies can be employed:

Selective modulation: Instead of completely blocking EPCR, fine-tune its signaling by using selective inhibitors or modulators. This approach enhances beneficial effects while minimizing harmful ones.

Combination therapies: Utilize EPCR-targeted therapies alongside anticoagulants, anti-thrombotics, or anti-inflammatory drugs that target different pathways, as well as therapies that promote vascular integrity.

Tissue-specific delivery: Employ targeted delivery methods, such as nanoparticles, to specifically administer EPCR modulators to affected tissues, such as RA synovial joints.

The efficacy and safety of EPCR-targeted therapies will depend on the targeting method, dosage, duration, and individual patient variability. Therefore, thorough preclinical and clinical studies are essential.

## 7. Conclusions

EPCR emerges as a pivotal factor in the complex pathophysiology of RA, impacting both inflammatory and coagulation processes. Given that RA is marked by elevated inflammation and a greater risk of thromboembolic and cardiovascular incidents, the significance of EPCR becomes evident. Targeting EPCR presents a promising therapeutic strategy to reduce inflammation, enhance endothelial function, mitigate the aggressiveness of RASF, and lower the risk of thrombosis and CVD in RA patients. Several approaches, including recombinant APC, APC analogs, monoclonal antibodies, and combination therapies, offer exciting opportunities for improving disease management. Understanding the balance between soluble and membrane-anchored EPCR may also provide insights into patient stratification and personalized treatment strategies. Future research is essential to elucidate how EPCR influences RA pathogenesis and to validate therapeutic strategies targeting this receptor in clinical settings. By expanding our understanding of EPCR’s diverse roles, we can potentially harness its therapeutic potential to improve outcomes for individuals with RA.

## Figures and Tables

**Table 1:** Haplotypes of the *PROCR* gene and their frequencies in healthy controls and the mutations associated effects.

Haplotype (H)	Frequency	Rare Allele Tagged	Related Protein Level	Effects
**H1**	~40%	4678G/C (rs9574) [17]	↓sEPCR ↑APC [22]	↓VTE [22], pregnancy loss [23]. ↑Early onset ischemic stroke [24] and Kawasaki disease [25].
**H2**	~43%	No [17]	No changes	Standard function
**H3**	10–21%	4600A>G (rs867186) [17]	↑sEPCR [15], PC and FVII [17] ↓mEPCR [26]	↑VTE [27], pregnancy loss [23], coronary heart disease [28], and breast cancer [29]. ↓Severe malaria in adults [30].
**H4**	~5%	3811G/A [17]	↑sEPCR slightly [31]	↑VTE slightly [31]

APC: activated protein C; FVII: factor VII; mEPCR: membrane-anchored endothelial protein receptor; PC: protein C; sEPCR: soluble EPCR; VTE: venous thromboembolism.

**Table 2:** EPCR ligands and their binding initiated functions.

Ligand	Functions When Bound to EPCR
**Activated protein C** **Protein C (APC/PC)**	Enhances the generation and prolongs APC’s presence [39]. Promotes APC’s anticoagulant, cytoprotective, and anti-inflammatory effects [40].
**Antiphospholipid antibodies**	Promotes pro-inflammatory and pro-thrombotic effects [12].
**Factor VII**	Initiates the coagulation [41] and anti-inflammatory effects [42].
**Factor X**	Regulates its interaction with the TF-FVIIa complex [43]. Promotes endothelial barrier function [44].
**Plasmodium falciparum erythrocyte membrane protein 1**	Promotes adhesion of parasite-infected erythrocytes to the endothelium, avoiding their clearance by the spleen [45]. Causes severe inflammation and tissue damage in malaria [45].
**Proteinase-3/Macrophage-1**	Binds to activated neutrophils [46]. Is associated with leukocyte extravasation [47].
**Sphingosine-1-Phosphate**	Mediates APC’s suppressing role in osteoclast differentiation [48]. Protects the endothelial barrier [49].
**Secretory Phospholipase A2 Group V**	Promotes inflammation and the invasiveness of RASF [50]. Disrupts the endothelial barrier [51].
**T cell receptor**	Triggers γδ T cells response to stress induced by infection or malignancy [36].

TF: Tissue factor; RASF: rheumatoid arthritic synovial fibroblasts.

## Data Availability

Data sharing is not applicable to this article as no datasets were generated or analyzed during the current study.
